# Supplementary figures and images for: How to use frailtypack for validating failure-time surrogate endpoints using individual patient data from meta-analyses of randomized controlled trials
Source: PLoS One. 2020 Jan 28;15(1):e0228098. doi: 10.1371/journal.pone.0228098 (PMC6986733; doi:10.1371/journal.pone.0228098)

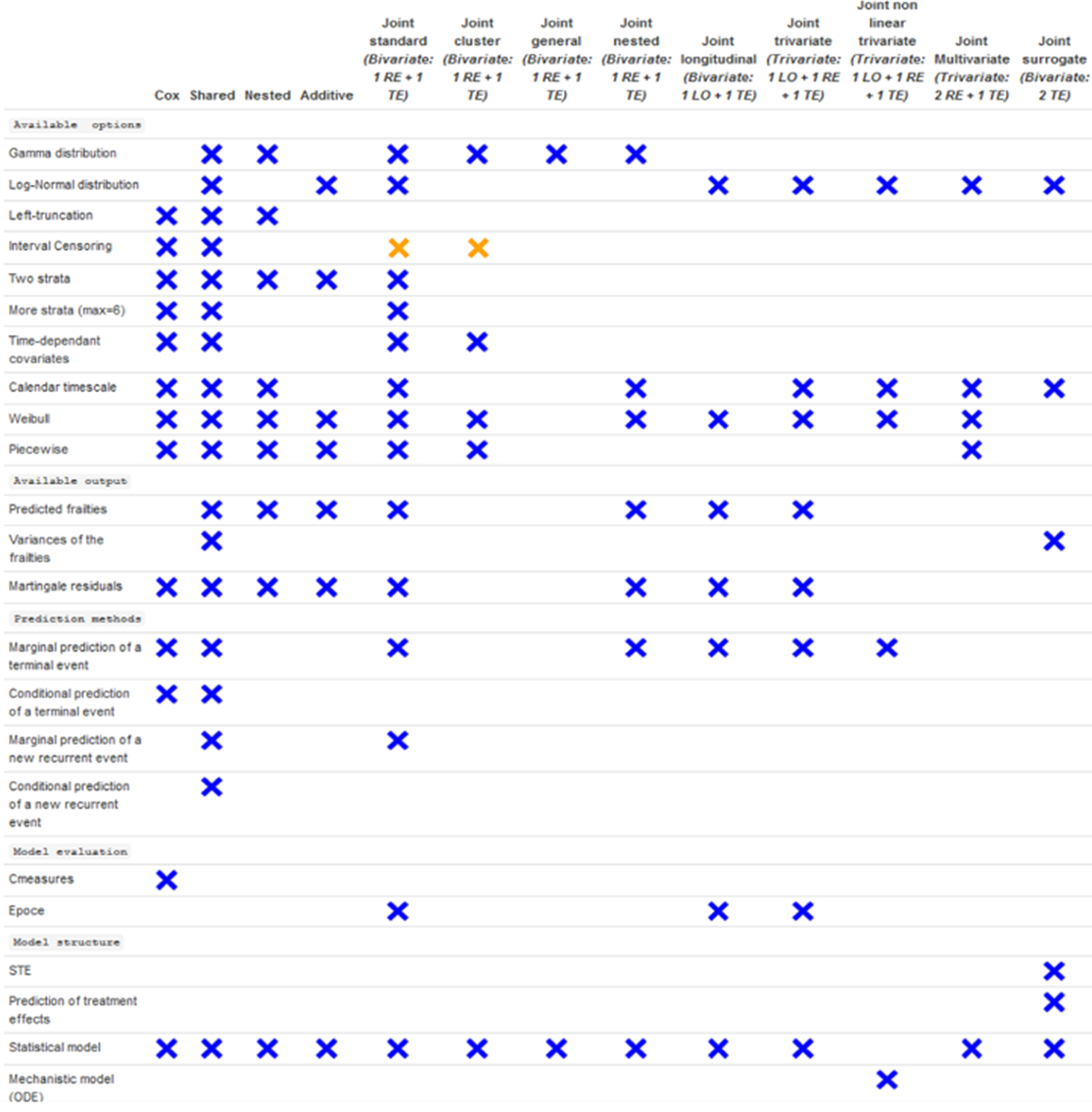

Supplement: S1 Fig — Blue cross is for the option available for a given type of model in the package on CRAN, orange cross is for the option included in the package but not yet on CRAN yet. Empty cells mean that an option is not available for a given type of model. RE = Recurrent Event. TE = Terminal Event. LO = Longitudinal Outcome. STE = Surrogate Threshold Effect. ODE = Ordinary Differential Equation. (TIF) [file pone.0228098.s001.tif]
