# Supplementary material for: How to use frailtypack for validating failure-time surrogate endpoints using individual patient data from meta-analyses of randomized controlled trials
Source: PLoS One. 2020 Jan 28;15(1):e0228098. doi: 10.1371/journal.pone.0228098 (PMC6986733; doi:10.1371/journal.pone.0228098)
Supplement: S1 Appendix — (PDF) [file pone.0228098.s002.pdf]

S1 Appendix: Extension of the methodology

S1A Appendix. Penalized likelihood approach

The baseline hazard functions $\hat{\lambda}_{0S}(.)$ and $\hat{\lambda}_{0T}(.)$ are approximated using a linear combination of Cubic M-splines and polynomial functions of 3rd order [1]. The cumulative hazard functions are approximated using I-splines (integrated M-splines). This allows smoothing functions, which is useful in epidemiology. In the semi-parametric approach, regardless of the expected smooth baseline hazard functions, the likelihood of the model is penalized by a term depending on the roughness of the functions [2]. Therefore, the penalized marginal log-likelihood is given by:

$$pl(\Phi) = l(\Phi) - \kappa_1 \int_0^\infty \lambda_{0S}^{''2}(t)dt - \kappa_2 \int_0^\infty \lambda_{0T}^{''2}(t)dt \qquad (.1)$$

where $l(\Phi)$ is the full marginal log-likelihood. κ_1 and κ_2 are positive smoothing parameters which control the trade-off between the data fit and the smoothness of the functions. The smoothing parameters are chosen using a maximizing likelihood cross-validation criterion described in Joly *et al.* [2], based on two separate Cox proportional hazard models with no covariates. An alternative is to set κ_1 and κ_2 manually

S1B Appendix.Individual-level surrogacy

To measure the strength of the association between S_{ij} and T_{ij} after adjusting the marginal distributions for the trial and the treatment effects, we defined Kendall's τ as follows:

$$\begin{aligned}\tau \;=\; & 2\int_{u_i}\int_{\omega_{ij}}\int_{u_{i'}}\int_{\omega_{i'j'}} \\ & \frac{\exp(\omega_{ij}+u_i+\zeta\omega_{ij}+\alpha u_i)+\exp(\omega_{i'j'}+u_{i'}+\zeta\omega_{i'j'}+\alpha u_{i'})}{(\exp(\omega_{i'j'}+u_{i'})+\exp(\omega_{ij}+u_i))(\exp(\zeta\omega_{i'j'}+\alpha u_{i'})+\exp(\zeta\omega_{ij}+\alpha u_i))} \\ & \frac{1}{\sqrt{2\pi\theta}}\exp\left[-\frac{1}{2}\frac{\omega_{i'j'}^2}{\theta}\right]\frac{1}{\sqrt{2\pi\gamma}}\exp\left[-\frac{1}{2}\frac{u_{i'}^2}{\gamma}\right]d\omega_{i'j'}du_{i'} \\ & \frac{1}{\sqrt{2\pi\theta}}\exp\left[-\frac{1}{2}\frac{\omega_{ij}^2}{\theta}\right]\frac{1}{\sqrt{2\pi\gamma}}\exp\left[-\frac{1}{2}\frac{u_i^2}{\gamma}\right]d\omega_{ij}du_i - 1\end{aligned}$$

where θ, ζ, α and γ are estimated using the model (1). Kendall's τ is the difference between the probability of concordance and the probability of discordance of two realizations of S_{ij} and T_{ij}. It belongs to the interval [-1,1] and assumes a zero value when S_{ij} and T_{ij} are independent. We estimate it using Monte-Carlo or Gauss-Hermite quadrature integration methods, and parametric bootstrapping for confidence interval.

S1C Appendix. Trial-level surrogacy

The key reason for validating a surrogate endpoint is to be able to predict the effect of treatment on the true endpoint, based on the observed effect of treatment on the surrogate endpoint. As shown by Buyse *et al.*

[3], the coefficient of determination obtained from the covariance matrix Σ_v of the random effects treatment-by-trial interaction can be used to evaluate underlined prediction, and therefore as surrogacy evaluation measurement at trial level. It is defined by:

$$R^2_{trial} = \frac{\sigma^2_{v_{ST}}}{\sigma^2_{v_S}\sigma^2_{v_T}} \quad (.2)$$

The SEs of R^2_{trial} is calculated using the Delta method [4]. We also propose R^2_{trial} and 95% CI computed using parametric bootstrapping. Use of the Delta method can lead to confidence limits violating the [0,1], as noted by Burzykowski *et al.* [5]. However, using other methods would not significantly alter the findings of the surrogacy assessment.

S1D Appendix. Derivation of the surrogate threshold effect

Assume the distribution of the trial-specific treatment effects observed on the surrogate and true endpoints :

$$\begin{pmatrix}\beta_{S_i}\\ \beta_{T_i}\end{pmatrix} \sim MVN\left(\beta, \Sigma_v\right), \; with \quad \beta = (\beta_S, \beta_T)^\top, \quad and \quad \Sigma_v = \begin{pmatrix}\sigma^2_{v_S} & \sigma_{v_{ST}}\\ \sigma_{v_{ST}} & \sigma^2_{v_T}\end{pmatrix},$$

where β and Σ_v represent the fixed treatment effects and the variance-covariance matrix described in model (1). Let $i = 0$ the new trial for which data are available on the surrogate endpoint but not on the true endpoint. If β_{S_0} represent the observed treatment effect in trial 0 from a Cox model and ϑ represents the fixed-treatment effects parameters and variance components related to model (1), as shown in [3], the conditional mean of the treatment effect in trial 0, $\beta_T + v_{T0}$ can be written as

$$E(\beta_T + v_{T_0}|\beta_{S_0}, \vartheta) = \beta_T + \frac{\sigma_{v_{ST}}}{\sigma_{v_{SS}}}(v_{S_0} - \beta_S), \quad (.3)$$

with the conditional variance:

$$Var(\beta_T + v_{T0}|\beta_{S_0}, \vartheta) = Var(v_{T0})(1 - R^2_{trial}), \quad (.4)$$

where R^2_{trial} is defined in (.2), and v_{T_0} is the random effect treatment-by-trial interaction associated with the true endpoint. In practice, ϑ and β_{S_0} are unknown and have to be estimated. Model (1) can be fitted to the data to estimate ϑ, and a Cox proportional hazard model can be fitted to the data from the new trial to estimate β_{S_0}. The corresponding estimates may be denoted by $\hat{\vartheta}$ and $\hat{v}_{S_0}$. Therefore, the formulation of the prediction variance is:

$$Var(\beta_T + v_{T0}|\beta_{S_0}, \vartheta) \approx f\{Var(\hat{\beta}_{S_0})\} + f\{Var(\hat{\vartheta}\} + Var(v_{T0})(1 - R^2_{trial}), \quad (.5)$$

where $f\{Var(\hat{\beta}_{S_0})\}$ and $f\{Var(\hat{\vartheta}\}$ are functions of the asymptotic variance-covariance matrices of $\hat{v}_{S_0}$ and $\hat{\vartheta}$. These functions describe the contribution to the variability due to the use of the estimates of these parameters. Assume the simple case where the estimation errors are present in the meta-analysis but not in

the new trial. This assumption requires an infinite sample size for the new trial. In this case, $f\{Var(\hat{\beta}_{S_0})\}$ can be reduced to 0, and (.5) therefore can be written as:

$$Var(\beta_T + v_{T0}|\beta_{S_0}, \vartheta) \approx f\{Var(\hat{\vartheta}\} + Var(v_{T0})(1 - R^2_{trial}). \tag{.6}$$

Let $x = (1, -\sigma_{v_{ST}}/\sigma_{SS})^\top$. Given that in linear mixed-effects models the maximum likelihood estimates of the covariance parameters are asymptotically independent of the fixed effects parameters, one can rewrite the prediction variance (.7) as a quadratic function of β_{S_0} [6] as:

$$Var(\beta_T + v_{T0}|\beta_{S_0}, \vartheta) \approx x^\top \left[V_\mu + \left(\frac{\beta_{S_0} - \beta_S}{\sigma_{v_{SS}}} \right)^2 V_D \right] x + \sigma_{v_{TT}}(1 - R^2_{trial}), \tag{.7}$$

where V_μ and V_D are the asymptotic variance-covariance matrices of $(\hat{\beta}_T, \hat{\beta}_S)^\top$ and $(\hat{\sigma}_{v_{ST}}, \hat{\sigma}_{v_{SS}})^\top$, respectively. The limits of the $(1 - \gamma)100\%$ prediction interval for $\beta_T + v_{T0}$ are functions of v_{S_0}, and correspond to:

$$l(\beta_{S_0}) \equiv E(\beta_T + v_{T0}|\beta_{S_0}, \vartheta) - z_{1-(\gamma/2)} \sqrt{Var(\beta_T + v_{T0}|\beta_{S_0}, \vartheta)} \tag{.8}$$

for the lower prediction limit function, and

$$u(\beta_{S_0}) \equiv E(\beta_T + v_{T0}|\beta_{S_0}, \vartheta) + z_{1-(\gamma/2)} \sqrt{Var(\beta_T + v_{T0}|\beta_{S_0}, \vartheta)}, \tag{.9}$$

for the upper prediction limit function. In (.8)-(.9), $z_{1-(\gamma/2)}$ is the $(1-(\gamma/2))$ quantile of the standard normal distribution. Assume that $\sigma_{v_{ST}} > 0$ and that negative values of α_i indicate a beneficial treatment effect in trial i. One can compute a value of β_{S_0} such that the upper prediction limit

$$u(\beta_{S_0}) \equiv 0. \tag{.10}$$

This value represents the STE. The solution(s) of equation (.10) can be obtained by solving a quadratic equation. The number of solutions of the equation depends on the configuration of the parameters of $u(\beta_{S_0})$. In the event of two solutions, STE is the lower value. Therefore, an observed value of treatment effect on a surrogate endpoint higher than STE predicts a significant treatment effect on the true endpoint. Note that there may be two possible values for STE depending on the variance used to compute $u(\beta_{S_0})$. Thus, STE can be influenced by the prediction variance, and then by the characteristics of the meta-analysis, and the new trial.

S1E Appendix. Interpretation of STE and decision-making

A large value of STE would point to the need to observe a large treatment effect on the surrogate endpoint in order to conclude in a non-zero treatment effect on the true endpoint. If the STE is high, it would not be reasonable to use the surrogate [6], even if it were potentially valid (with $R^2_{trial} \approx 1$).

The IQWiG suggest basing the prediction of the treatment effect on the true endpoint both on the treatment effect observed on the surrogate endpoint, on R^2_{trial} and on STE [7]. The use of STE is mainly

required in the event of a moderate correlation with 0.7 < R < 0.85 ($0.49 < R^2_{trial} < 0.72$). In this case, if the upper bound of the 95% CI (or the upper bound of the 80% CI) of the treatment effect on the surrogate is lower than STE, there is at most an indication of an effect on the true endpoint. If the correlation is high (R ≥ 0.85 or $R^2_{trial} \geq 0.72$), and if there is a treatment effect on the surrogate, there is at most an indication of an effect on the true endpoint.

References

[1] J. O. Ramsay, Monotone regression splines in action, Statist. Sci. 3 (4) (1988) 425–441. doi:10.1214/ss/1177012761.
URL https://doi.org/10.1214/ss/1177012761

[2] P. Joly, D. Commenges, L. Letenneur, A penalized likelihood approach for arbitrarily censored and truncated data: Application to age-specific incidence of dementia, Biometrics 54 (1) (1998) 185–194.
URL http://www.jstor.org/stable/2534006

[3] M. Buyse, G. Molenberghs, T. Burzykowski, D. Renard, H. Geys, The validation of surrogate endpoints in meta-analyses of randomized experiments, Biostatistics 1 (1) (2000) 49–67.

[4] B. E. Dowd, W. H. Greene, E. C. Norton, Computation of standard errors, Health Services Research 49 (2) (2014) 731–750.
URL http://doi.org/10.1111/1475-6773.12122

[5] T. Burzykowski, G. Molenberghs, M. Buyse, H. Geys, D. Renard, Validation of surrogate end points in multiple randomized clinical trials with failure time end points, Journal of the Royal Statistical Society C (Applied Statistics) 50 (4) (2001) 405–422. doi:10.1111/1467-9876.00244.
URL http://dx.doi.org/10.1111/1467-9876.00244

[6] T. Burzykowski, M. Buyse, Surrogate threshold effect: An alternative measure for meta-analytic surrogate endpoint validation, Pharmaceutical Statistics 5 (3) (2006) 173–186. doi:10.1002/pst.207.
URL http://dx.doi.org/10.1002/pst.207

[7] Institute for Quality and Efficiency in Health Care, Validity of surrogate endpoints in oncology: Executive summary (2011).
URL www.iqwig.de/download/A10-05_Executive_Summary_v1-1_Surrogate_endpoints_in_oncology.pdf
