# Supplementary material for: How to use frailtypack for validating failure-time surrogate endpoints using individual patient data from meta-analyses of randomized controlled trials
Source: PLoS One. 2020 Jan 28;15(1):e0228098. doi: 10.1371/journal.pone.0228098 (PMC6986733; doi:10.1371/journal.pone.0228098)
Supplement: S2 Appendix — (PDF) [file pone.0228098.s003.pdf]

S2 Appendix: Description of the arguments and return values for the functions

S2A Appendix. The `jointSurroPenal()` function

Description:. The mandatory argument of this function is `data`, which must be a dataframe containing at least seven columns named:

- `patienID:` a numeric, that represents the patient's identifier and must be unique;
- `trialID:` a numeric, that represents the trial in which each patient was randomized;
- `timeS:` the follow up time associated with the surrogate endpoint;
- `statusS:` the event indicator associated with the surrogate endpoint, with 0 = no event, 1 = event;
- `timeT:` the follow up time associated with the true endpoint;
- `statusT:` the event indicator associated with the true endpoint, with 0 = no event, 1 = event;
- `trt:` the treatment indicator for each patient, with 1 = treated, 0 = untreated.

Argument `maxit` indicates the maximum number of iterations to be reached by the Marquardt algorithm, the default being `40`. To simplify the model, it is possible to set the coefficients α or ζ to `1` and not to estimate them, or to assume homogeneity on the baseline hazard functions. Arguments `indicator.zeta` and `indicator.alpha` are binary and indicates whether ζ and α should be estimated (1) or not (0). These parameters could be set to `0` in the event of convergence and identification issues. Argument `frail.base` indicates whether the heterogeneity between trials on the baseline risk using the shared cluster specific frailties (u_i) should be considered (`1`), or not (`0`).

Argument `n.knots` indicates the number of knots between 4 and 20, which corresponds to `n.knots + 2` splines functions for approximating the baseline hazard functions (the same number of hazard functions for both endpoints). This value is required in the penalized likelihood estimation. The convergence thresholds of the Marquardt algorithm are for the difference between the consecutive values of estimated coefficients (`LIMparam`), the difference between two consecutive penalized log-likelihoods (`LIMlogl`) and for the small gradient of the log-likelihood (`LIMderiv`). By default, these thresholds values are set to 10^{-3}.

The log-likelihood formulation associated with the joint model (1) includes two integration levels which cannot be solved analytically. Argument `int.method` is a numeric which indicates the integration method to be used for approximating integrals over the random effects. When this parameter is set to `0`, the full Monte Carlo integration method is used; if set to `1` the full Gauss-Hermite quadrature is used; if set to `2`, a combination of both Gauss-Hermite quadrature for integration over the individual-level random effects and Monte Carlo for integration over the trial-level random effects is used; if set to `4`, a combination of both Monte Carlo to integrate over the individual-level random effects and Gauss-Hermite quadrature to integrate over the trial-level random effects is used. In the event of Gauss-Hermite quadrature integration, argument

`adaptatif` is used to indicate whether the pseudo adaptive Gauss-Hermite quadrature `(1)` [1] or the classical Gauss-Hermite quadrature `(0)` should be used. Argument `nb.gh` indicates the number of nodes to be used in the Gauss-Hermite quadrature integration method and can be 5, 7, 9, 12, 15, 20 or 32. Argument `nb.gh2` is the number of nodes for the Gauss-Hermite quadrature in the event of convergence issues to re-estimate the model. Argument `nb.iterPGH` indicates the number of iterations before the re-estimation of the posterior random effects, in the event of the two-steps pseudo-adaptive Gauss-Hermite quadrature [2]. If this parameter is set to `0`, there is no re-estimation. With the Monte-Carlo integration method, argument `nb.mc` is used to indicate the number of samples considered, a value between 100 and 300 normally giving good results. However, beyond 300, the program takes a long computing time to obtain estimates.

By default, the integrals in Kendall's τ formulation are approximated using the Monte-Carlo integration. Argument `nb.MC.kendall` indicates the number of generated samples used in this case. It is advisable to use at least 4000 samples for stable results. Argument `nboot.kendall` specifies the number of samples considered in the parametric bootstrap to estimate the confidence interval of Kendall's τ, or R^2_{trial}. The default is `1000`.

Argument `true.init.val` is numeric with two values, `0` or `2`, that indicate how to consider initial values of model parameters. If set to `(0)`, initial values given to the parameters will be considered. If `true.init.val` is set to `2`, α and γ are initialized using two different shared frailty models [3]; $\sigma^2_{v_S}$, $\sigma^2_{v_T}$ and $\sigma_{v_{ST}}$ are set by the user; ζ, θ, β_S and β_T are initialized using a classical joint frailty model, considering individual level random effects [4]. If the joint frailty model is encounters to convergence issues, β_S and β_T are initialized using two shared frailty models. In all other scenarios, if the simplified model does not converge, default values are used. Initial values for the associated parameters of splines are set to `0.5`. Arguments `theta.init`, `sigma.ss.init`, `sigma.tt.init`, `sigma.st.init`, `gamma.init`, `betas.init`, `betat.init`, `alpha.init`, `zeta.init` are vectors of initial values for variances of random effects, regression coefficients, and α and ζ parameters. By default, θ, α and ζ are initialized to `1`, σ^2_S, σ^2_T, γ, $beta_S$, β_T to are initialized to `0.5`, and σ^2_{ST} are initialized to `0.48`.

As other arguments of this function, `scale` is a numeric that makes it possible to re-scale survival times and to avoid numerical problems for some convergence issues. If no change is needed the argument is set to 1, the default value. e.g.: 365 aims to convert days to years ".

Argument `init.kappa` is a vector of two smoothing parameters used to penalized the log likelihood. By default, `init.kappa = NULL` meaning that the values used should be obtained using the maximizing likelihood cross-validation criterion [5]. Argument `kappa.use` is a numeric with values `1`, `3` or `4` that indicate how to manage the smoothing parameters κ_1 and κ_2 in the event of convergence issues. If it is set to `1`, the given smoothing parameters or those obtained by cross-validation are used. If it is set to `3`, the associated smoothing parameters are successively divided by 10, in the event of convergence issues until 5 times. If it is set to `4`, the management of the smoothing parameter is as in case `1`, followed by the successive division as described in case `3` and preceded by the change of the number of nodes for the Gauss-Hermite quadrature. The default is `4`.

When the program requires a random number generator, argument `random. generator` indicates the random number generator to use by the `Fortran` compiler with a value `1` for the intrinsec subroutine `Random_number` and `2` for the subroutine `uniran()`; argument `random` is a binary that indicates wether the random number generator should be reset with a different environment at each call `(1)` or not `(0)`. if `random` is set to `1`, the computer clock is used as seed and it will not be possible to reproduce the generated data (or dataset); argument `random` is required if `random.generator` is set to 1. Argument `random.nb.sim` is a binary that indicates the number of generations that will be made, required if both `random.generator` and `random` are set to 1. The parameter `seed` is the seed to use for data (or samples) generation, required if `random.generator` is set to 1. `seed` must be a positive value. Otherwise, the program will not consider it.

The other arguments for this function are `nb.decimal`, which indicates the number of decimals required for presenting results, `print.times`, a logical that specifies wether the estimation time should be printed or not, and `print.iter`, which is a logical that specifies wether the iteration process should be printed or not. The default values for the last two arguments are `FALSE`.

The `jointSurroPenal` object:. value `EPS` is a vector containing the convergence thresholds obtained with the Marquardt algorithm for the parameters for the log-likelihood and for the gradient. Vector `b` refers to the estimates of the spline parameters, the coefficient ζ (if `indicator.zeta` is set to `1`), the standard error of the shared individual-level frailty ω_{ij} (θ), elements of the lower triangular matrix (L) from the Cholesky decomposition such that $\Sigma = LL^T$, with Σ the covariances of the random effects (v_{S_i}, v_{T_i}), the coefficient α (if `indicator.alpha` is set to `1`), the standard error of the random effect u_i and the regression coefficients β_S and β_T. Value `varH` is the covariance matrix of all parameters in `b` and `varHIH` is the corresponding robust estimation of the covariance matrix of estimates. Value `loglikPenal` represents the penalized log-likelihood. Value `LCV` represents the approximated likelihood cross-validation criterion (see equation .1), with H^{-1} the converged Hessian matrix and `l(.)` the full log-likelihood.

$$LCV = \frac{1}{n}(trace(H_{pl}^{-1}H) - l(.)) \tag{.1}$$

Value `xS` represents a vector of times where both survival and hazard functions are estimated; `survS`, `lamS`, `lamT` and `survT` are four arrays `(dim = 3)` for estimates and confidence bounds of: baseline survival and hazard function for surrogate endpoint, baseline survival and hazard function for true endpoint. `n.iter` is the number of iterations used to reach convergence. The `'jointSurroPenal'` object also contains estimates of distinct parameters of the model labeled `theta` for θ, `gamma` for γ, `alpha` for α `zeta` for ζ, `sigma.s` for σ_S, `sigma.t` for σ_T, `sigma.st` for σ_{ST}, `beta.s` for β_S and `beta.t` for β_T.

Other returned values include: `ktau` and `R2.boot` which represent Kendall's τ and R^2_{trial} with the corresponding 95 % CI computed using the parametric bootstrap; `Coefficients` which is a matrix of the estimates with the corresponding standard errors and the 95 % CI; and `kappa` which represents a vector of positive smoothing parameters used for convergence. These values may be different from the initial values if argument `kappa.use` is set to `3` or `4`; `data`, the dataset used in the model; and `varcov.Sigma`, which is the covariance

matrix of ($\hat{\sigma_S}$,$\hat{\sigma_T}$, $\sigma_{\hat{ST}}$) obtained from the Delta method. The latter is used to predict the treatment effect on the true endpoint as well as in the computation of the surrogate threshold effect (STE).

available functions associated with the 'jointSurroPenal' object. For any object inherited from the class 'jointSurroPenal', the following R functions are available:

- `jointSurroTKendall()`: used to compute Kendall's τ;
- `summary()`: used to display the results of the surrogacy evaluation, based on model (1);
- `ste()`: used to compute the surrogate threshold effect;
- `loocv()`: used for the leave-one-out cross-validation, in order to assess the accuracy of the prediction using the model (1) ;
- `predict()`: this function allows for new trials to predict the treatment effect on the true endpoint based on the observed treatment effect on the surrogate;
- `plot()`: used to plot the baseline survival and the baseline hazard functions.

By calling `help()` on each of the above functions, one can obtain an exhaustive description of its use.

S2B Appendix. The `jointSurrSimul()` function

The first two arguments refer to the size of the meta-analysis (or the multicenter study) be considered, and include the total number of subjects represented by `n.obs` and the total number of trials (or centers), represented by the `n.trial` parameter. The fixed censorship time should be specified using the argument `cens.adm`. The desired model parameters should be specified in the arguments `theta` for the variance of the shared frailty term at the individual level (θ), `alpha` for the coefficient α, `gamma` for the variance of the shared frailty term at the trial level associated with the baseline hazard (γ), `zeta` for the coefficient ζ, `sigma.s` and `sigma.t` for the variances of the correlated random effects treatment-by-trial interaction (σ_S^2, σ_T^2). The desired level of correlation between v_{S_i} and v_{T_i} is given by the argument `rsqrt`, in such a way that the corresponding coefficient of determination $R^2_{trial} = rsqrt^2$. Arguments `betas` and `betat` represent the fixed treatment effects associated with the surrogate endpoint (β_S) and with the true endpoint (β_T).

Using the `jointSurrSimul()` function, it is possible to generate the dataset by considering homogeneous baseline hazard functions across trials. In this case, the argument `frailt.base` should be set `0`. To consider heterogeneity, this parameter should be set to `(1)`, i.e. the default. The desired parameters for the Weibull hazard function are given by the arguments `lambda.S` as scale parameter and `nu.S` as shape parameter, which are both associated with the Surrogate endpoint; `lambda.T` and `nu.T` as the scale and shape parameters associated with the True endpoint. The argument `ver` indicates the number of covariates to be considered in the joint model. To evaluate surrogacy, we just consider the treatment arm. Argument `typeOf` is used to

indicate the variant of the joint model used for data generation. It is coded `0` for the classical joint model with a shared individual frailty effect [4] and `1` for the joint surrogate model (1). If `typeOf` is set to `0`, arguments `gamma`, `zeta`, `sigma.s`, `sigma.t` and `rsqrt` are not required.

The parameter `equi.subj.trial` is a binary variable that indicates wether the same proportion of subjects should be included per trial (1) or not (0). If set to 0, the proportions of subject per trial are required in the argument `prop.subj.trial` (a vector of `n.trial` elements). Likewise, argument `equi.subj. trt` is a binary variable that indicates if the same proportion of subjects should be randomized per trial (1) or not (0). If set to 0, the proportions of subject per trial are required in the argument `prop.subj.trt`. The argument `full.data` with required values 0 or 1 indicates the structure of the returned dataset.

The other arguments of this function are assigned to the random data generator. Arguments `random.generator` , `random`, `random.nb.sim` and `seed` are detailed in the function `jointSurroPenal()` . Argument `nb.reject.data` specifies the number of generated datasets to be rejected before the considered one. This parameter is required in the event of data generation for simulation studies. With the same values and `random.generator` set to 1, all generated dataset will be the same. However, if the argument `nb.reject.data` varies, different datasets will be obtained during the data generation process.

If the argument `full.data` is set to 0, the function `jointSurrSimul()` returns a `dataframe` with the same columns as those detailed in the function `jointSurroPenal()`. However, if `full.data` is set to 1, additional columns corresponding to the random effects ω_{ij}, u_i, v_{S_i} and v_{T_i} are included in the `dataframe`. Note that in the latter case, the presence of u_i, v_{S_i} and v_{T_i} requires `typeOf` to be equal to `1`.

S2C Appendix. The `jointSurroPenalSimul()` function

Description:. argument `nb.dataset` indicates the number of datasets (or meta-analysis) to be generated. It can be set between 100 and 500. Arguments `nbSubSimul` and `ntrialSimul` specify respectively the number of subjects and the number of trials to be considered in each meta-analysis. Argument `equi.subj.trial` is a binary that indicates whether the same proportion of subjects per trial should be considered in each study design (1), the default or not (0). In the event of different trial sizes, put the proportions of subjects to be considered per trial in the vector `prop.subj.trial`. Argument `equi.subj.trt` indicates whether the same proportion of subjects randomized in the treatment arm should be considered per trial `(1)`, the default or not `(0)`. If `0`, put the proportions of subjects to be considered per trial in `prop.subj.trt`. The sizes of the vectors `prop.subj.trial` and `prop.subj.trt` are equal to the number of trials. Arguments `theta2`, `gamma.ui`, `sigma.s`, `sigma.t`, `betas`, `betat`, `zeta`, `alpha.ui` are simulation values for variances of random effects (θ, γ, σ^2_S, σ^2_T), regression coefficients (β_S, β_T) , and ζ and α parameters. The desired trial level association (R^2_{trial}) is specified using argument `R2`. Users can implement the `R` function `jointSurroKendall()` to set the desired Kendall's τ by varying the values assigned to the arguments α, γ, ζ and θ. Recall that Kendall's τ formulation depends on these variables.

Arguments `lambdas`, `lambdat`, `nus` and `nut` are desired scale and shape parameters for the `Weibull`

distribution associated with the Surrogate endpoint and with the True endpoint. Argument `time.cens` indicates the censorship time. The default for `time.cens` is `549` for about 40% of censored subjects, taking into account the Weibull parameters.

Argument `true.init.val` is a numeric used to manage initial values during estimations, with values `0`, `1` or `2`. If it is set to `(0)` (the default), models should be initialized using arguments `theta.init` for θ, `zeta.init` for ζ, `gamma.init` for γ, `alpha.init` for α, `sigma.ss.init` for $\sigma^2_{v_S}$, `sigma.tt.init` for $\sigma^2_{v_T}$, `sigma.st.init` for $\sigma_{v_{ST}}$, `betas.init` for β_S and `betat.init` for β_T. If `true.init.val` is set to `1`, the real simulation parameters are used as initial values. If set to `2`, α and γ are initialized using two distinct shared frailty models [3]; $\sigma^2_{v_S}$, $\sigma^2_{v_T}$ and $\sigma_{v_{ST}}$ are set by the user using the previous corresponding initial values; ζ, θ, β_S and β_T are initialized using the classical joint frailty model, considering individual level random effects [4]. If the joint frailty model is faced with convergence issues, β_S and β_T are initialized using two shared frailty models. In all other scenarios, default parameters values are used if the simplified model does not converge. Initial values for spline associated parameters are set to `0.5`.

Argument `kappa.use` is a numeric with values `0`, `1`, `2`, `3` or `4`, that indicates how to manage the smoothing parameters κ_1 and κ_2 in the event of convergence issues. If it is set to `0`, the first smoothing parameters that allowed convergence on the first dataset are used for all simulations. If it is set to `1`, smoothing parameters are estimated by cross-validation for each generated dataset. If it is set to `2`, the same process for choosing kappas as in case `1`. However, in the event of a convergence issue, the first smoothing parameters that allowed convergence among the three previous that worked are used. If it is set to `3`, the associated smoothing parameters are successively divided by 10, in the event of convergence issues until 5 times. If it is set to `4` (the default), the management of the smoothing parameters is as in case `2`, preceded by the successive division described in case `3` and by the changing of the number of nodes for the Gauss-Hermite quadrature.

The other arguments are detailed in the function `jointSurroPenal()`. Argument `random.nb.sim` can be set to `nb.dataset`.

The `jointSurroPenalSimul` object:. the function `jointSurroPenalSimul()` returns an object from the class `'jointSurroPenalSimul'` of which all elements can be obtained by displaying the help on this function. Some of the returned values include: `n.iter`, which is the mean number of iterations needed to reach converge; `dataTkendall` and `dataR2boot`, which are two matrices with `nb.dataset` line(s) and three columns of the estimates of Kendall's τ and R^2_{trial} with their confidence intervals; and `dataParamEstim`, which is a dataframe including all estimates with the associated standard errors for all simulations. All cases of non-convergence in previous matrices and data frames are represented by a line of 0.

S2D Appendix. The `jointSurroTKendall()` function

Argument `object` is an object inherited from the `'jointSurroPenal'` class. This argument can be set to `NULL` if users want to base computation on given parameters of Kendall's τ function. In that case, argument

`theta` indicates the variance of the individual-level random effect (ω_{ij}); `gamma`, the variance of the trial-level random effect associated with the baseline risk (u_i); `alpha`, the coefficient associated with u_i; `zeta`, the coefficient associated with ω_{ij}. A argument `sigma.v` is for the covariance matrix of the random effects treatment-by-trial interaction (v_{S_i}, v_{T_i}).

Argument `int.method` is a numeric that indicates the integration method. If `int.method` is set to `0`, the Monte-Carlo integration is used with the number of generated samples specified using argument `nb.MC.kendall`. However, if `int.method` is set to `1`, the Gauss-Hermite quadrature integration is used with the number of nodes specified using argument `nb.gh`. For better or stable results, one should use at least 4000 samples or `20` quadrature nodes. The actual value for nodes used is the maximum between `20` and `nb.gh`.

Argument `ui` is a binary that indicates wether heterogeneity at trial level associated with the baseline risk should be considered (`1`), the default or not (`0`). The other arguments of the function `jointSurroPenal()` are detailed in the function `jointSurroPenal()`.

S2E Appendix. The `plot()` function

In this function, arguments `x` is an object inherited from the `'jointSurroPenal'` class. Argument `endpoint` is a binary that indicates the endpoint to be plotted: `0` for the surrogate endpoint, `1` for the true endpoint, and `2` for both endpoints, the default. Argument `scale` is a numeric that allows survival times to be re-scaled. If no change is needed, the argument is set to 1, the default value. Otherwise, the survival times are multiplied by the scale value. Argument `type.plot` is a character string specifying the type of curve to be plotted. Possible values are "Hazard", or "Survival". It is possible to simply use the first letters "Haz" and "Su". Argument `conf.bands` is a logical that determines whether confidence bands will be plotted. Arguments `xmin` and `xmax` indicate the minimum and the maximum values for the x-axis, and `ylim` the range of the y-axis. The location of the legend in the graph can be specified by setting the argument `pos.legend` to a single keyword from the list `"bottomright"`, `"bottom"`, `"bottomleft"`, `"left"`, `"topleft"`, `"top"`, `"topright"`, `"right"` and `"center"`. Argument `cex.legend` allows the size of the legend to be changed. The last three arguments: `main`, `Xlab` and `Ylab` are labels for the title of the plot, for the x-axis and for the y-axis.

S2F Appendix. The `loocv()` function

Principle and description:. for each trial i, the loocv consists of the following: (a) estimating the parameters of model (1) using the dataset minus subjects in trial i; (b) predicting the treatment effect on the true endpoint based on both the observed treatment effect on the surrogate endpoint in trial i and the estimates from (a); (b) comparing the observed treatment effect on the true endpoint, in trial i with the predicted value. In (a) and (b), the observed treatment effects on the surrogate and the true endpoints are estimated

using two Cox proportional hazard models. The function `loocv()`, which can be applied to an object of class `'jointSurroPenal'`, has three main arguments.

Argument `object` is an object inherited from class `'jointSurroPenal'`; `unusedtrial` is an argument that specifies a list of trial(s) not to be considered in the cross-validation. This argument is useful when excluding some trials, when the model is facing a convergence issue. The last argument is `var.used` and has two values. The first one is `"error.estim"` and indicates whether the prediction variance takes the estimation errors from the estimates of the parameters into account. If estimates are supposed to be known, or if the dataset includes a sufficiently high number of trials with a sufficiently high number of subjects per trial, value `"No.error"` can be used. The default is `error.estim`.

S2G Appendix. Management of convergence issues

Special attention must be paid to initializing model parameters, the choice of the number of knots per spline, the smoothing parameters and the number of quadrature points for solving convergence issues. When numerical or convergence problems are encountered, the model is fitted again using a combination of the following strategies: varying the number of quadrature points, dividing or multiplying κ_1 or κ_2 by 10 or 100 according to their preceding values, or using parameter vectors obtained during the last iteration (with a modification of the number of quadrature points and smoothing parameters). Using this strategy, a lower rejection rate is usually obtained during simulation studies. A sensitivity analysis was conducted without this strategy and similar results were obtained on the converged samples with about 23% rejection rate.

References

[1] D. Rizopoulos, Fast fitting of joint models for longitudinal and event time data using a pseudo-adaptive gaussian quadrature rule, Computational Statistics & Data Analysis 56 (3) (2012) 491 – 501. doi:https://doi.org/10.1016/j.csda.2011.09.007.
URL http://www.sciencedirect.com/science/article/pii/S0167947311003264

[2] L. Ferrer, V. Rondeau, J. Dignam, T. Pickles, H. Jacqmin-Gadda, C. Proust-Lima, Joint modelling of longitudinal and multi-state processes: Application to clinical progressions in prostate cancer, Statistics in Medicine 35 (22) (2017) 3933–3948. doi:10.1002/sim.6972.
URL https://onlinelibrary.wiley.com/doi/abs/10.1002/sim.6972

[3] V. Rondeau, D. Commenges, P. Joly, Maximum penalized likelihood estimation in frailty models, Lifetime Data Analysis 9 (2) (2003) 139–153.

[4] V. Rondeau, S. Mathoulin-Pelissier, H. Jacqmin-Gadda, V. Brouste, P. Soubeyran, Joint frailty models for recurring events and death using maximum penalized likelihood estimation: Application on cancer events, Biostatistics 8 (4) (2007) 708–721. doi:10.1093/biostatistics/kxl043.
URL http://biostatistics.oxfordjournals.org/content/8/4/708.abstract

[5] P. Joly, D. Commenges, L. Letenneur, A penalized likelihood approach for arbitrarily censored and truncated data: Application to age-specific incidence of dementia, Biometrics 54 (1) (1998) 185–194. URL http://www.jstor.org/stable/2534006
