# Supplementary material for: How to use frailtypack for validating failure-time surrogate endpoints using individual patient data from meta-analyses of randomized controlled trials
Source: PLoS One. 2020 Jan 28;15(1):e0228098. doi: 10.1371/journal.pone.0228098 (PMC6986733; doi:10.1371/journal.pone.0228098)
Supplement: Attachment — Submitted filename: Response to Reviewers.pdf [file pone.0228098.s005.pdf]

PONE-D-19-20769R1

How to use *frailtypack* for validating failure-time surrogate endpoints using individual patient data from meta-analyses of randomized controlled trials

We thank the editor and the reviewers for the great interest to our manuscript. Please find below our replies to the reviewers' comments. We have also revised the manuscript in accordance with the comments when necessary.

6. Review Comments to the Author

Please use the space provided to explain your answers to the questions above. You may also include additional comments for the author, including concerns about dual publication, research ethics, or publication ethics. (Please upload your review as an attachment if it exceeds 20,000 characters)

Reviewer #1: I would like to thank the authors for their thorough responses to reviewers' comments - I think the manuscript has greatly improved, and my previous comments have been mostly addressed. Well done.

However, I still have some (minor) comments:

IQWiG is first introduced in line 111, but the acronym is introduced only in line 199 - I would move the acronym to line 111 instead.

Done

There is some style inconsistency: some R output is coloured, some is not. Please make consistent throughout the manuscript, I am not sure if this will be copy-edited later on.

Thank you to reviewer #1 for this comment. Actually, only the R codes used for the outputs are colored. The uncolored codes are related to the definition (or description) of the corresponding functions. This is the case for all functions in section "Available functions in the frailtypack R package for surrogacy evaluation" and the S3method plot() in section "Illustration". We chose not to color the outputs.

Line 287: it is a bit confusing what figure are you referring to. Please reword the sentence.

We reworded the sentence as:

"A list of other models implemented in frailtypack [23] can be found in S1 Fig"

In computational details and package installation: it is not necessary to specify the repos argument when installing a package. Furthermore, the {devtools} functionality to install packages from GitHub seems to have been moved to the {remotes} package (and only imported in {devtools}). I would consider referring to {remotes} rather than {devtools}, but this is completely up to you.

You are right concerning the points mentioned above. However, the repos argument helps to guide the user on the repository that we are currently used to install the package and to avoid on some platforms (such as in a Linux terminal) the selection in a proposed list the repository to use. Moreover, the use of this argument does not negatively impact the functionality of the command. Regarding the use of the package "remotes" rather than "devtools", we have not experienced the first one and as mentioned by the reviewer, this package is imported in "devtools". So, we chose to use the "devtools" package since it allows several tools necessary for the compilation of the package.

I just noticed that the column with the patient ID is assumed to be "patienID". Should it be "patientID" instead? This would be more coherent with the other names, where the whole word is used.

We rewrote the column name patienID of argument data as patientID. We updated the package and the manuscript subsequently

In the section on choosing model via the LCV: I think it is still a bit difficult to compare the estimates of the two competing models. I think it might be helpful to include a separate table that compares them side by side. Besides that, I still think that the coefficients are not so similar - including the confidence intervals that show large overlap would be great too, and would sell the point better (in my opinion).

We thank reviewer #1 for this comment. We aim in this section to illustrate how to use LCV for model comparison. It is not really the goal to compare the estimates of the two competing models, given that they can be different. So by using LCV, one can choose the best model according to the data. We rephrased the interpretation in the corresponding section as follows:

"As expected [17], the two observed values of LCV are quite similar. The summary() function applied to previous objects give results shown below. When comparing the two models, estimates of most coefficients and standard errors showed some differences. However this observation does not alter conclusions on the surrogacy validity captured by Kendall's tau and R2trial."

Some sentences are a bit confusing at the moment, please reword:

Lines 365-366;

We rephrased the sentence as:

“By changing the value of some arguments, we obtained the following set of arguments/values which allowed convergence:”

Line 382: “observation on...” could be replaced by “the estimated value of…”;

Done

Line 389: reword after the comma;

We rephrased the sentence as:

“*These parameters can be interpreted as usual, but taking adjustment on the random effects into account*.”

Lines 508-510.

We rephrased the sentence as:

“By varying the values of the arguments in the *jointSurroPenal* function, convergence of the model is not always guaranteed. Therefore, it is important in the event of convergence issues to know how to play with the arguments/values couple as shown in the previous section.”

Finally, I found some more typos:

Line 103, replace “random effect” with “random effects”

Done

Lines 380-381, replace “compare” with “compared”

Done

Line 552, replace “neccesary” with “necessary”

Done

Reviewer #2: Much more details had provided in the revision, and the involvement of professional typewriter improved the readability noticeably. The revision addressed all my concerns.

Thank you
